# Extracranial metastases of anaplastic meningioma

**DOI:** 10.1259/bjrcr.20150092

**Published:** 2017-02-07

**Authors:** Robbin Zachery Thomas, Ishani Dalal

**Affiliations:** Radiology, Henry Ford Hospital, Detroit, MI, Michigan

## Abstract

Anaplastic meningioma is a World Health Organization (WHO) Class III lesion representing 2–3% of all meningiomas, with more aggressive spread, increased mortality and increased likelihood of recurrence. Metastases outside the blood–brain barrier are uncommon but can occur to the lungs, liver, bone and skin.Definitive diagnosis is obtained with pathological analysis. The World Health Organization classifies meningiomas into benign (Grade I), atypical (Grade II) and anaplastic/malignant (Grade III) based on histological findings including number of mitoses, cellularity, nucleus to cytoplasm etc. This case presents a 58-year-old female with history of treated anaplastic meningioma with new onset headache, nausea and vomiting. Workup demonstrated multiple new bilateral pulmonary nodules, which subsequent biopsy proved to be metastasis from recurrent anaplastic meningioma, with extensive intrathoracic involvement.

## Clinical vignette

The patient is a 58-year-old female with past medical history of left breast cancer status post resection and chemotherapy (2000), basal cell carcinoma of the forehead (1997) and pathology proven anaplastic intracranial meningioma (9/2012) status post three surgical resections, radiation therapy and radiosurgery with ongoing antiangiogenic therapy with bevacizumab, who presented to the emergency department in mid-2014 with headache, nausea and vomiting. Acute abdominal series demonstrated multiple new bilateral pulmonary nodules. Subsequent CT revealed both pulmonary and pleural components, and MRI of the brain demonstrated recurrent brain mass in area of previous surgical resection (fFigure 1). Whole body positron emission tomography/CT demonstrated avid fludeoxyglucose uptake with significant SVU within nodules involving the lungs, pleura and epiphrenic/retrocrural lymph nodes (Figure 2). Subsequent left pleural biopsy revealed spindle cell neoplasm with pathology similar to findings from previous intracranial resection specimen. Immunohistochemical staining results included focal dot-like positive epithelial membrane antigen (EMA) stain, high MIB-1 (Mindbomb E3 ubiquitin protein ligase 1) proliferation index up to 45% in more active areas, glial fibrillary acidic protein (GFAP) staining suggestive of multifocal areas of invasion of the brain, negative CD34 ruling out the possibility of solitary fibrous tumour and negative S-100 staining (Figures 3 and 4). Octreotide was added to the patient's regimen at that time. Several months later, the patient again presented with left rib pain and shortness of breath with CT demonstrating progression of large pleural and parenchymal metastatic disease. Subsequent positron emission tomography/CT confirmed lung, chest wall, pleural, chest and upper abdomen lymph node and osseous involvement of a thoracic vertebral body and right iliac bone. The patient transitioned to palliative care and has since passed.

**Figure 1.**
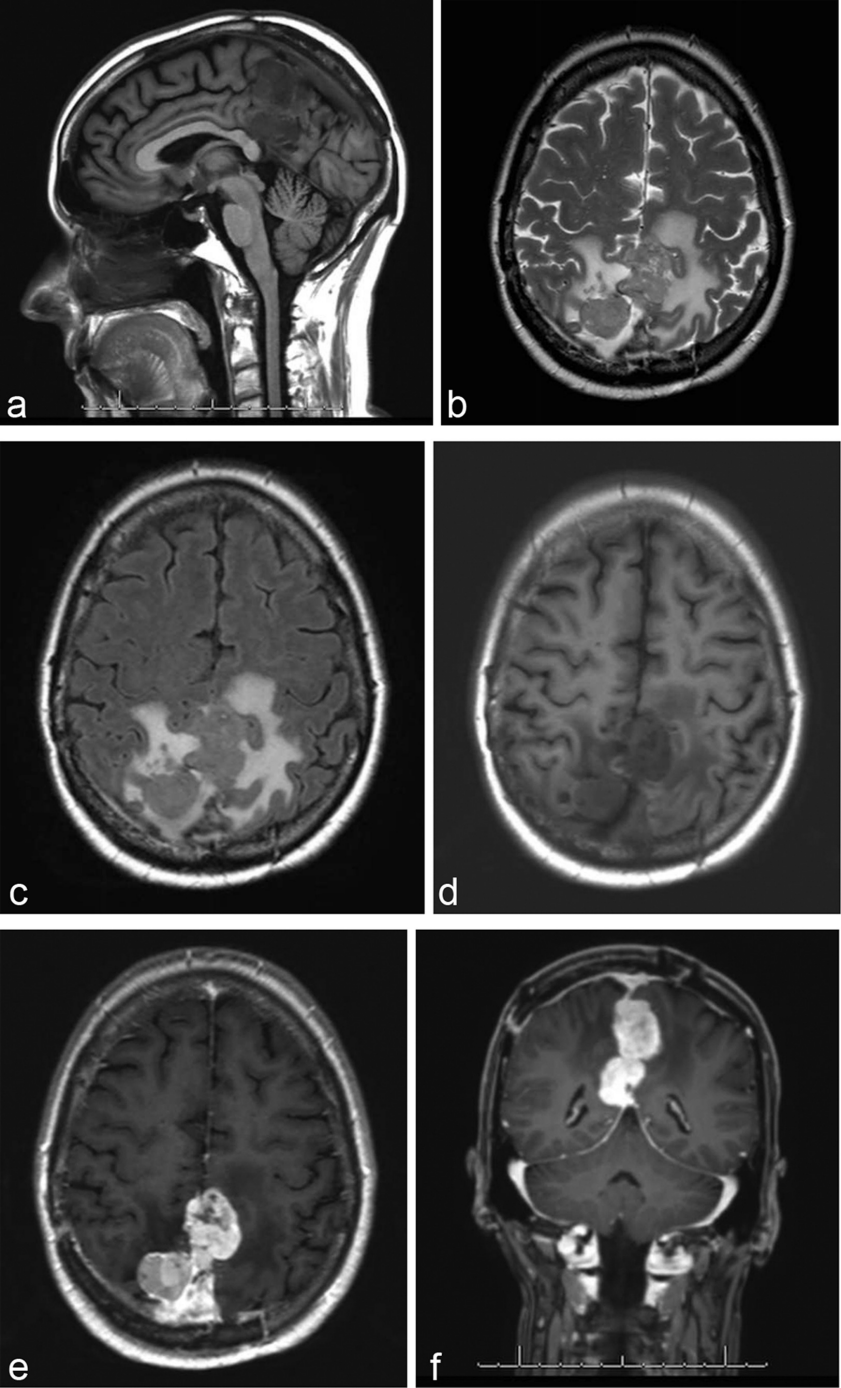
MRI with and without contrast of the brain, top row left to right sagittal *T*_1_, axial *T*_2_, fluid-attenuated inversion-recovery and bottom row left to right axial *T*_1_ precontrast, axial *T*_2_ postcontrast, coronal *T*_1_ postcontrast. MRI demonstrates recurrent multilobulated heterogeneous mass with associated oedema and avid enhancement. There is extension into the falx-tentorial region and likely involvement of the superior sagittal and straight sinuses. There is mass effect on the corpus callosum posteriorly.

**Figure 2.**
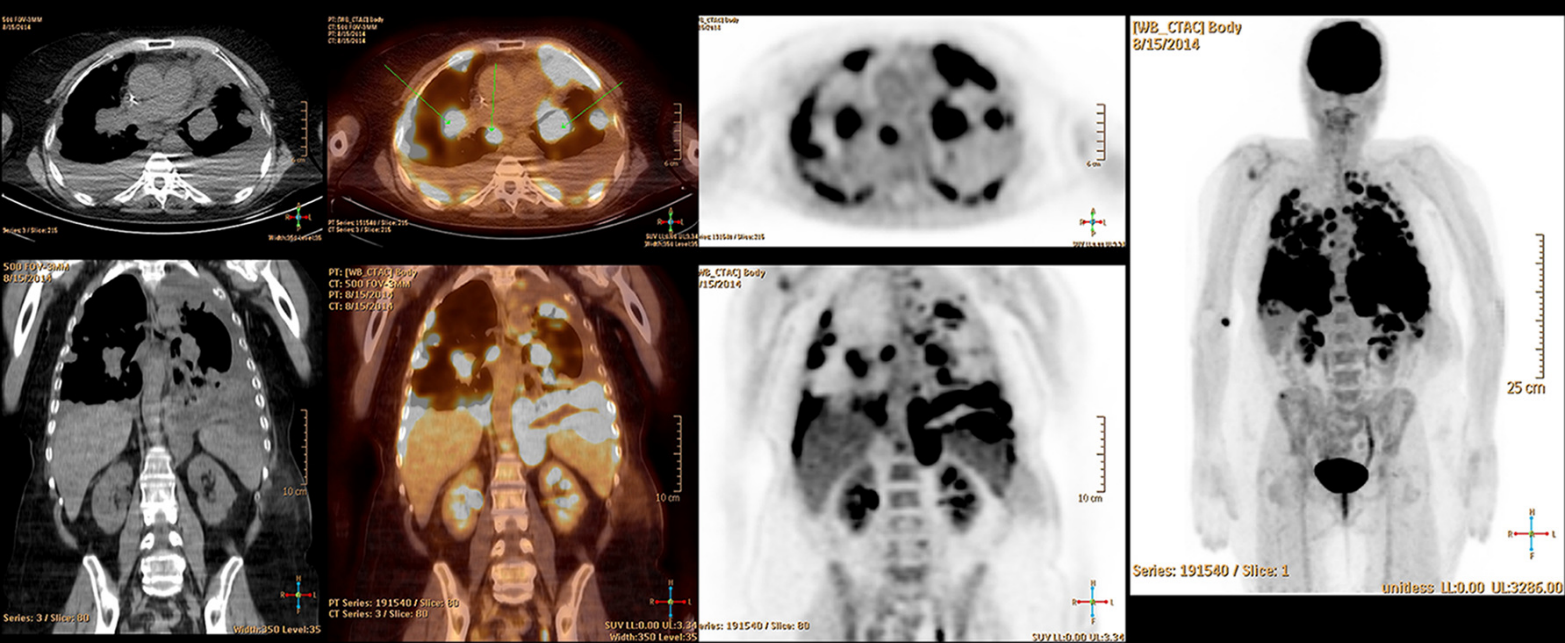
Positron emission tomography-CT obtained within 1 year of figure 1 demonstrates multiple foci of abnormal fludeoxyglucose uptake in both lungs and pleura, which correspond to conglomerate masses on concurrent CT. Maximum SUV = 11.1. Also noted is extensive involvement of hilar, mediastinal, retrocrural and upper abdomen lymph nodes as well as partially visualized *T*_12_ vertebral body involvement. Right iliac bone involvement is not seen on provided images. Left pleural biopsy resulted in spindle cell neoplasm with pathology similar to intracranial tumour resection specimen and previous intracranial tumour resection specimen (not included).

**Figure 3.**
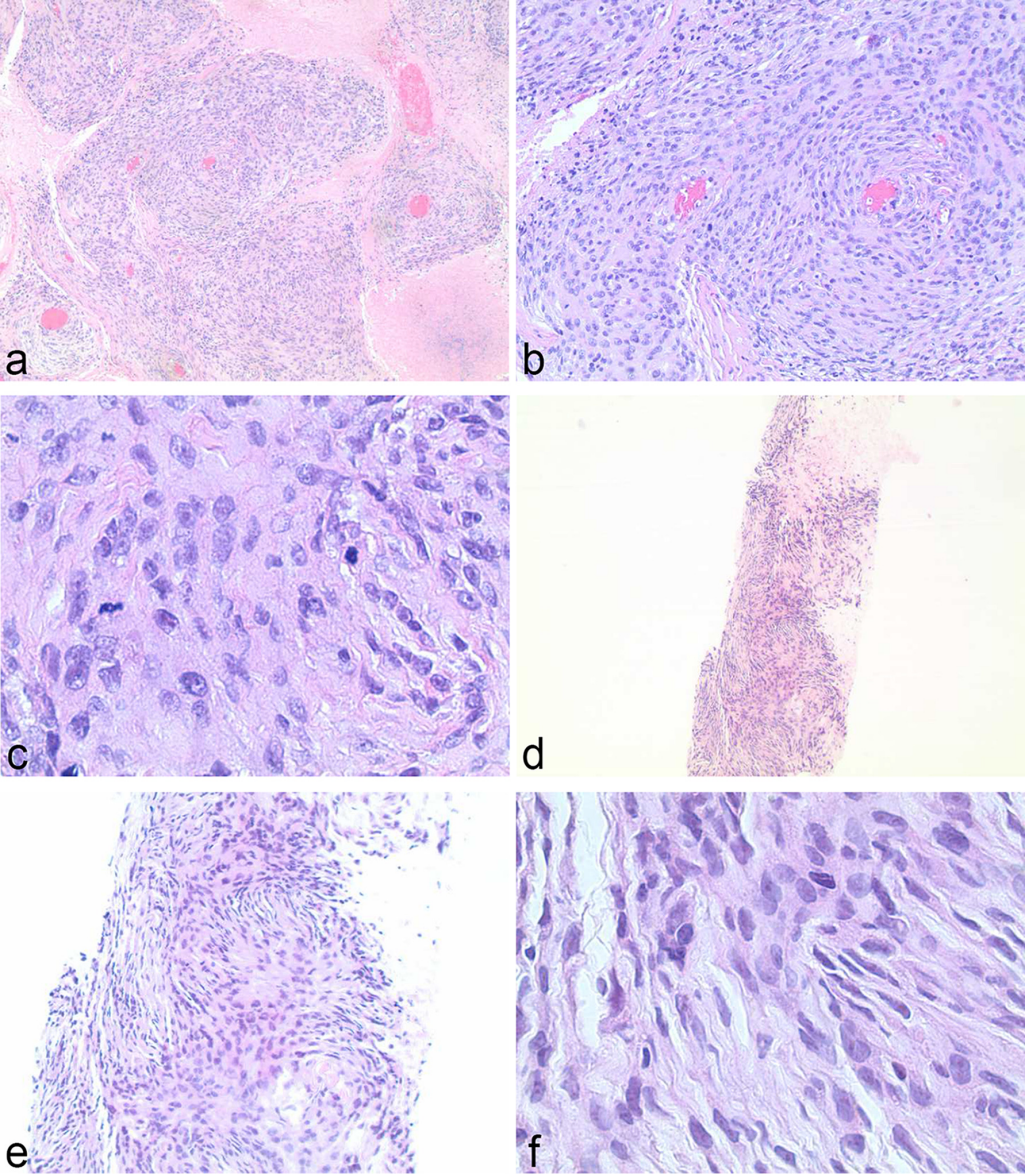
Haematoxylin and eosin stained histologic slides of left pleural soft tissue nodule biopsy sample, with top row left to right 4×, 10×, and 40× magnification of resected intracranial mass demonstrating proliferation of spindled meningothelial cells with an excess of 35 mitoses per 10 HPF (high power field), compatible with anaplastic meningioma (WHO 3). Bottom row left to right 4×, 10× and 40× magnification of left pleural nodule biopsy consistent with low to intermediate grade malignant spindle cell neoplasm with similar morphological appearance to resected intracranial meningioma.

**Figure 4.**
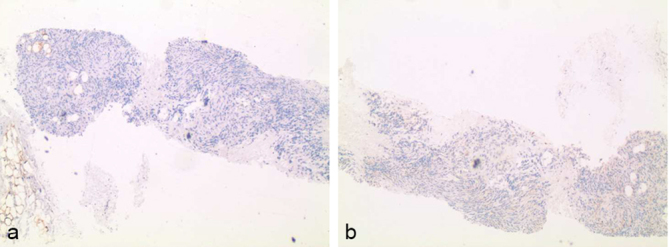
Immunohistochemical staining of left pleural nodule biopsy sample with epithelial membrane antigen (top left) demonstrates focal weak dot-like positive stain. Epithelial membrane antigen stain is a typical marker of meningioma, with decreased expression often seen with anaplastic meningioma and recurrent meningioma. Staining with S-100 (bottom right) is negative, consistent with meningioma.

## Background

Meningiomas arise from arachnoid cap cells of the meninges and represent approximately 13–26% of intracranial tumours.^1^ They are often found incidentally and can present with symptoms relating to size and location such as headache, nausea, vomiting, seizure, visual deficits, hearing loss or weakness. Anaplastic meningiomas represent 2–3% of all meningiomas and demonstrate more aggressive characteristics with increased mortality and likelihood of recurrence. Metastases outside the blood–brain barrier are uncommon but can occur to the lungs, liver, bone and skin.^1,2^ Atypical and anaplastic meningiomas have been suggested in some studies to represent a malignant progression of benign meningiomas, with some series suggesting up to 28.5% of recurrent benign meningiomas demonstrating atypical or anaplastic pathology.^2,3^

## Epidemiology and prognosis

The annual prevalence rate for meningiomas is approximately 6 per 100,000. The majority of meningiomas occurs in middle aged or elderly patients but can also be seen in younger populations with syndromes such as neurofibromatosis Type 2.^2^ Benign meningiomas are more prevalent in females, whereas atypical and anaplastic meningiomas are more commonly seen in men.^2^ There are some reports of atypical meningiomas after cranial irradiation in younger populations with intracranial tumours as well as implication of dental x-rays or past low-dose experimental treatments for issues such as tinea capitas.^4^ Radiation-induced meningiomas are more likely to involve multiple sites, to recur early after excision and have a higher tendency to involve osseous structures.^5^

A tumour suppressor gene on chromosome 22q12 has been implicated in the initiation of meningioma formation and is seen in up to 70% of surgical specimens.^6^ Factors such as male sex, age less than 40 years and meningioma location at the skull base or parasagittal-falcine area are believed to be associated with increased recurrence rate after subtotal resection in anaplastic meningiomas.^1,7^ Prognosis is poor after development of recurrent disease as there is high likelihood of treatment failure.^4^ Median survival time for benign meningiomas with brain invasion is between 10 and 14 years with a 5 year mortality rate of 25%. Median survival time for anaplastic lesions without brain invasion is 1.5 years with a 5-year mortality rate of 68%, but median survival time is reduced to 1.4 years with a 5-year mortality rate of 83% in the presence of brain invasion.^8^

## Imaging

CT characteristics such as heterogeneous precontrast examination with homogenous bright enhancement after contrast injection, associated bone destruction, lack of calcification, indistinct margins and nodular contours are not unique to more aggressive variants and may also be seen in benign meningiomas. Similar remarks can be made about MRI findings such as indistinct margins on *T*_1_ weighted images or post-contrast *T*_1_ enhancement pattern.^2^ A future role for apparent diffusion coefficient (ADC) mapping and diffusion tensor imaging may be considered in non-invasive differentiation between benign and atypical and anaplastic meningioma with studies showing significantly higher ADC values and more disorganized water motion in benign meningiomas. Other studies suggest a role of magnetic resonance spectroscopy in differentiating higher grade meningiomas by their increases in choline/creatinine ratio, lactate and methylene.^9,10^

## Pathology

Anaplastic meningiomas are thought to be formed either de novo or via transformation of a pre-existing meningioma.^11^ The World Health Organization (WHO) classifies meningiomas into benign (Grade I), atypical (Grade II) and anaplastic/malignant (Grade III) based on histological findings including number of mitoses, cellularity, nucleus-to-cytoplasm ratio etc. Criteria for anaplastic meningioma includes “>/=mitotic figures/10 HPF or focal or diffuse loss of meningothelial differentiation resulting in carcinoma-, sarcoma-, or melanoma –like appearance”.^12^

Immunohistochemical findings that can help differentiate meningioma from other central nervous system neoplasms such as hemangiopericytoma and schwannoma include positive EMA, weakly positive S-100 staining and strong vimentin staining, which often stays positive through the various WHO grades.^13^ Decreased EMA positivity, as seen with the presented case, can occur with recurrence or malignant progression to Grade III anaplastic meningioma.^11^ S-100 immunoreactivity is not universally seen with meningiomas, as with our case, but when present, is often less reactive than would be expected when sampling schwannoma or solitary fibrous tumour.^13^ Ki-67/MIB-1 is used as an indication of cellular proliferation, and was found to be up to 45% in the more active areas of the presented case specimen. Ki-67-MIB-1 proliferative index has been found to increase from Grade I to Grade III meningiomas in a large retrospective study performed by Roser et al,^14^ with mean values of 3.88%, 9.95% and 12.18%, respectively.

Studies by Perry et al^7,8^ found microscopic brain invasion to be the most powerful predictive tool for disease recurrence. Independently, maximal mitotic rate of >/=4/10 HPF was found to increase the chance of recurrence. Frank histologic anaplasia, defined by the Mayo Clinic scheme as >/=20 mitotic figures/10 HPF or loss of meningothelial differentiation with or without brain invasion, is noted as the worst prognostic finding.

## Treatment

Surgical resection is the primary treatment for all grades of meningioma, with complete excision associated with decreased recurrence and increased survival.^8^ Anaplastic tumours are often found to adhere to cortical vessels, making resection difficult and increasing the risk of postoperative complications (e.g. infarction, oedema). Hypervascular tumours not supplied by the internal carotid artery may be treated with embolization prior to surgical resection to shrink tumour volume and potentially lower blood loss.^15^ Repeat surgical resection is commonly performed with disease recurrence. Early postsurgical adjunct therapy with conventional radiation has been shown to slow or halt disease progression, increase disease-free survival and increase overall survival in atypical and anaplastic meningiomas.^1^ Stereotactic radiosurgery is considered for lesions <3 cm following subtotal resection, recurrent disease, asymptomatic disease or in otherwise non-operative cases.^16^ Newer antiangiogenic therapies targeting vascular endothelial growth factor and receptors take advantage of a 10-fold increased expression of vascular endothelial growth factor in anaplastic meningiomas, with reports suggesting some delay in disease progression.^17^ Hydroxyurea has been shown to inhibit in vitro meningioma cell growth, with some response in benign meningiomas but equivocal response in anaplastic tumours. Therapies targeting the platelet-derived growth factor receptors are currently being investigated as these receptors are significantly increased in atypical and anaplastic meningiomas.^8^

## Summary

Anaplastic meningioma is a WHO Class III lesion with worse prognosis and increased recurrence rate after treatment as compared to Class I and Class II lesions. Definitive diagnosis is obtained with pathological analysis with ADC and diffusion tensor imaging characteristics on MRI showing promise in non-invasive differentiation.^9,10^ The case presented demonstrates a rare complication of anaplastic meningioma with extracranial metastatic disease. Extracranial metastasis most commonly spreads to the lungs, but can also be seen in the liver, bone and skin.^1,2^

## Learning points

1. Anaplastic meningioma is a WHO Class III lesion representing 2–3% of all meningiomas, with more aggressive spread, increased mortality and increased likelihood of recurrence.^1,2^
2. Metastases outside the blood–brain barrier are uncommon but can occur to the lungs, liver, bone and skin.^1,2^
3. CT characteristics such as heterogeneous precontrast examination with homogeneous bright enhancement after contrast injection, associated bone destruction, lack of calcification, indistinct margins and nodular contours, are not unique to more aggressive variants and may also be seen in benign meningiomas.^2^
4. Pathological criteria for anaplastic meningioma includes “>/=mitotic figures/10 HPF or focal or diffuse loss of meningothelial differentiation resulting in carcinoma-, sarcoma-, or melanoma –like appearance”.^12^
5. Surgical resection is the primary treatment for all grades of meningioma with adjunct therapy with conventional radiation shown to slow or halt disease progression, increase disease-free survival and increase overall survival in atypical and anaplastic meningiomas.^1,8^

## Consent

Written informed consent for the case to be published (including images, case history and data) was obtained from the patient(s) for publication of this case report, including accompanying images.
